# Jin-Gui-Shen-Qi Wan ameliorates diabetic retinopathy by inhibiting apoptosis of retinal ganglion cells through the Akt/HIF-1α pathway

**DOI:** 10.1186/s13020-023-00840-7

**Published:** 2023-10-12

**Authors:** Dan Liang, Yulin Qi, Lu Liu, Zhaoxia Chen, Shiyun Tang, Jianyuan Tang, Nianzhi Chen

**Affiliations:** 1 TCM Regulating Metabolic Diseases Key Laboratory of Sichuan Province, Hospital of Chengdu University of Traditional Chinese Medicine, Chengdu, China; 2 Department of Ophthalmology, The First Affiliated Hospital of Guangzhou University of Chinese Medicine, Guangzhou University of Chinese Medicine, Guangzhou, China; 3 College of Pharmacy, Chengdu University of Traditional Chinese Medicine, Chengdu, China; 4 Hospital of Chengdu University of Traditional Chinese Medicine, Chengdu, China; 5 State Key Laboratory of Ultrasound in Medicine and Engineering, College of Biomedical Engineering, Chongqing Medical University, Chongqing, China

**Keywords:** JGSQ, Diabetic retinopathy, Akt/HIF-1α pathway, Retinal ganglion cells

## Abstract

**Background:**

Jin-Gui-Shen-Qi Wan (JGSQ) has been used in China for thousands of years to treat various ailments, including frequent urination, blurred vision, and soreness in the waist and knees. It has traditional therapeutic advantages in improving eye diseases.

**Aim of the study:**

Clinical studies have confirmed the therapeutic efficacy of JGSQ in improving diabetes and vision; however, its efficacy and pharmacological effects in treating diabetic retinopathy (DR) remain unclear. Therefore, the aim of this study was to investigate the specific pharmacological effects and potential mechanisms of JGSQ in improving DR through a *db/db* model.

**Materials and methods:**

*db/db* mice were given three different doses of orally administered JGSQ and metformin for 8 weeks, and then PAS staining of the retinal vascular network patch, transmission electron microscopy, H&E staining, and TUNEL staining were performed to determine the potential role of JGSQ in improving DR-induced neuronal cell apoptosis. Furthermore, network pharmacology analysis and molecular docking were carried out to identify the main potential targets of JGSQ, and the efficacy of JGSQ in improving DR was evaluated through western blotting and immunofluorescence staining, revealing its mechanism of action.

**Results:**

According to the results from H&E, TUNEL, and PAS staining of the retinal vascular network patch and transmission electron microscopy, JGSQ does not have an advantage in improving the abnormal morphology of vascular endothelial cells, but it has a significant effect on protecting retinal ganglion cells from apoptosis. Through network pharmacology and molecular docking, AKT, GAPDH, TNF, TP53, and IL-6 were identified as the main core targets of JGSQ. Subsequently, through western blot and immunofluorescence staining, it was found that JGSQ can inhibit HIF-1α, promote p-AKT expression, and inhibit TP53 expression. At the same time, inhibiting the release of inflammatory factors protects retinal ganglion cells and improves apoptosis in DR.

**Conclusion:**

These results indicated that in the *db/db* DR mouse model, JGSQ can inhibit the expression of inflammatory cytokines and protect retinal ganglion cells from apoptosis, possibly by modulating the Akt/HIF-1α pathway.

**Graphical Abstract:**

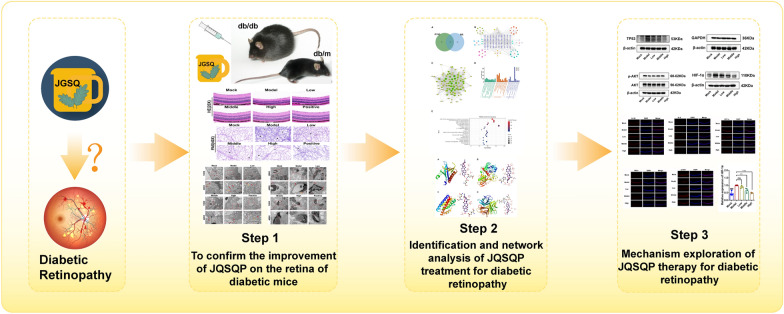

**Supplementary Information:**

The online version contains supplementary material available at 10.1186/s13020-023-00840-7.

## Introduction

Diabetic retinopathy (DR) is the most common, representative, and serious microvascular complication in patients with diabetes mellitus (DM) [1]. The incidence of diabetes is increasing annually, and it is estimated that by 2040, there will be approximately 642 million people with diabetes, among whom approximately 224 million people will develop DR, which is the most common reason for vision loss in diabetic patients [2, 3]. Clinical intervention with intensive glucose-lowering therapy, anti-inflammatory drugs such as glucocorticoids, and other treatments has obtained certain visual and anatomical prognoses for DR [4, 5]. However, a large amount of evidence from evidence-based medicine suggests that these therapies cannot prevent the occurrence of microvascular endpoints in diabetic patients. Therefore, actively exploring the biological targets and prevention strategies of DR has become a hot and difficult research topic worldwide.

The pathological basis of DR is the abnormal function and structure of retinal capillaries [6]. Research on DR has mostly focused on retinal capillary lesions, but most patients with DR have visual function abnormalities even before the appearance of microvascular lesions in the fundus [7]. Retinal ganglion cells or glial cells often undergo disease before the onset of microvascular disease in the retina, so it is particularly important to protect retinal ganglion cells in the early prevention of DR [8]. Research has found that the main way in which retinal ganglion cells undergo apoptosis is by transporting neurotrophic factors along the axons and axoplasm [9]. Ischaemia and hypoxia can lead to axoplasmic interruption of the optic nerve, activating apoptotic genes and inducing apoptosis in retinal ganglion cells [10, 11]. Nitric oxide also has neurotoxic effects on retinal ganglion cells. In the serum, vitreous humour, aqueous humour, and retina of patients with DR, inflammatory factors such as IL-1β, IL-6, and TNF-α are widely expressed [12]. Inhibiting the expression of inflammatory factors can delay or prevent the vascular genesis, neurodegenerative changes, and apoptosis of retinal ganglion cells in DR. Therefore, studying the preservation and maintenance of the physiological function of retinal ganglion cells in diabetic patients can effectively control the occurrence and development of DR. Among them, protecting retinal ganglion cells from apoptosis and regulating the ischaemic and hypoxic environment, as well as inhibiting inflammation, are reliable methods.

Jin-Gui-Shen-Qi Wan (JGSQ) is a traditional Chinese herbal formula from *Synopsis of the Golden Chamber* that has been used for thousands of years in China since the Eastern Han dynasty [13]. JGSQ is composed of *Rehmannia glutinosa* Libosch., *Dioscorea opposita* Thunb., *Cornus officinalis* Sieb. et Zucc., *Poria cocos* (Schw.) Wolf, *Paeonia suffruticosa* Andr., *Alisma orientalis* (Sam.) Juzep., *Aconitum carmichaelii* Debx. and *Cinnamomum cassia* Presl. It is widely used in clinical practice to treat various diseases, mainly to improve symptoms of reproductive, vision and urinary disorders as well as chronic asthma [14]. Many clinical studies and evidence-based medicine have found that JGSQ is safe for T2DM patients. Compared with hypoglycaemic agents alone, combination treatment with oral JGSQ enhances the effect on glucose metabolism in patients with T2DM [15]. In particular, clinical studies have confirmed that JGSQ can improve the retinal lesions of DR patients, with significant effects in improving retinal microaneurysms, oedema, and cotton-wool spots [16]. Therefore, JGSQ has the potential to improve diabetic complications, and further exploration is warranted for its therapeutic potential in improving DR.

In this study, a DR model was constructed using *db/db* mice, and JGSQ was administered orally for treatment. The protective effect of JGSQ on retinal ganglion cells in DR was confirmed by transmission electron microscopy, H&E staining, and TUNEL assays. Furthermore, network pharmacology analysis and molecular docking technology were used to identify the main target of JGSQ, and western blotting and immunofluorescence were used to further validate the mechanism of action of JGSQ in protecting retinal ganglion cells and improving apoptosis in DR. This study provides new therapeutic options for the prevention and treatment of DR.

## Materials and methods

### Preparation of JGSQ extract

 Rehmanniae Radix (cat: 2208041), Dioscoreae Rhizoma (cat: 2207115), Corni Fructus (cat: 2209074), Poria (cat: 2210033), Cortex Moutan (cat: 2209099), Alismatis Rhizoma (cat: 2210070), Radix Aconiti Lateralis (cat: 2210108) and Cortex Cinnamomi (cat: 2210074) purchased from Sichuan Xinhehua Traditional Chinese Medicine Decoction Pieces Co., LTD. Implementation standard: “Chinese Pharmacopoeia” 2020 edition. JGSQ extract is prepared from common Chinese medicinal herbs in a certain weight ratio (Table 1). The total amount of medicinal herbs used in this study was 540 g. The above medicinal materials were immersed in 50% ethanol 10 times at room temperature for 1 h, sonicated at 45 °C for 30 min, extracted twice, combined with the medicinal solution, filtered, concentrated to 2 g/mL under reduced pressure by a rotary evaporator at 50 °C, and stored in a refrigerator at 4 °C. The trait description and identification tests of the medicinal materials were all performed by pharmacist Zhaoxia Chen, a Quality Assurance Manager from Koda Pharmaceutics Co., Ltd. The preparation of a JGSQ clinical drug extract was entrusted to a GMP pharmaceutical company, Koda Pharmaceutics Co., Ltd., in accordance with the licence number approved by the Ministry of Health and Welfare of the Republic of China: DOH Manufacturing No. 028491.

**Table 1:** JGSQ medicinal material and doses

TCM materia medica	Botanical nomenclature	Specifications of decoction pieces	Amount (g)
Rehmanniae Radix	*Rehmannia glutinosa* Libosch	Steaming, slice	160
Dioscoreae Rhizoma	*Dioscorea opposita* Thunb	Slice	80
Corni Fructus	*Cornus officinalis* Sieb. et Zucc	Steaming with wine	80
Poria	*Poria cocos* (Schw.) Wolf	Chunk	60
Cortex Moutan	*Paeonia suffruticosa* Andr	Slice	60
Alismatis Rhizoma	*Alisma orientalis* (Sam.) Juzep	Stir-frying with salt-water, slice	60
Radix Aconiti Lateralis	*Aconitum carmichaelii* Debx	Slice	20
Cortex Cinnamomi	*Cinnamomum cassia* Presl	Silk	20
Total amounts			540

### Ultra high performance liquid chromatography‒Q exactive-mass spectrometry (UHPLC‒QE‒MS)

The samples were crushed with a mixer mill for 120 s at 50 Hz. One hundred milligrams of sample was added to 500 μL of extracted solution dissolved in 80% methanol containing 10 μg/mL internal standard. After 30 s of vortexing, the samples were homogenized at 45 Hz for 4 min and sonicated for 1 h in an ice water bath. After placing for 1 h at − 40 °C, the samples were centrifuged at 12000 rpm (RCF = 13,800 (× g), R = 8.6 cm) for 15 min at 4 ℃. The supernatant was carefully filtered through a 0.22 μm microporous membrane, and then 100 μL from each sample was pooled as QC samples. The samples were stored at − 80 °C until UHPLC‒MS analysis. LC‒MS/MS analysis was performed on an UHPLC system. The sample injection volume was set at 5 μL. The flow rate was set at 0.5 mL/min. The multistep linear elution gradient program was as follows: 0–11 min, 85–25% A; 11–12 min, 25–2% A; 12–14 min, 2–2% A; 14–14.1 min, 2–85% A; 14.1–15 min, 85–85% A; 15–16 min, 85–85% A. The negative (Additional file 1: Fig. S1A) and positive (Additional file 1: Fig. S1B) results are shown in the Additional file 1: Fig. S1.

### Animal studies and reagents

The *db/db* and *db/m* mice at 9–10 weeks of age were purchased from Jiangsu GemPharmatech Co., Ltd. and housed under standard laboratory conditions with free access to food and water. After one week of adaptation feeding, *db/db* mice were randomly divided into 5 groups (n = 10 per group) for treatment: (1) *db/db* + normal saline group (model group); (2) *db/db* + JGSQ (5 g/kg/day) group (low group); (3) *db/db* + JGSQ (10 g/kg/day) group (middle group); (4) *db/db* + JGSQ (20 g/kg/day) group (high group); and (5) *db/db* + metformin (200 mg/kg/day) group (positive group). Metformin was dissolved in distilled water and orally administered at a dose of 200 mg/kg/day for 8 weeks. The *db/m* mice were used as the normal control group (n = 10). JGSQ was orally administered daily for 8 weeks. Body weight and FBG were measured weekly. This experiment was approved by the Animal Research Ethics Society of Chengdu University of Traditional Chinese Medicine (Approval number: 2023KL-010). The blood glucose level in the serum of mice was tested using a blood glucose test kit (Cat: ml076792, mlbio, Shanghai, China).

### Haematoxylin–eosin (H&E) staining

After anaesthetizing the mice with intraperitoneal injection, the eyeballs with attached optic nerves were removed bilaterally. They were fixed in neutral formalin solution for 72 h, dehydrated with a gradient of ethanol concentrations, treated with xylene for transparency, embedded in paraffin, and sliced according to thickness standards. Subsequently, haematoxylin staining and eosin staining were performed separately. H&E staining was performed to observe changes in tissue structure under a light microscope.

### PAS staining of retinal vascular network patches

After fixing the optic cup in precooled 4% paraformaldehyde for 48 h, it was rinsed with running water and cut into a petal shape around the optic disc as the centre. The inner layer of the retina was separated and soaked in 0.15 mol/L pH 7.4 glycine buffer overnight after being rinsed with PBS. Then, it was digested with 3% trypsin at 37 °C for 2 h. After the retina was dissolved, it was gently shaken in distilled water to wash away the inner limiting membrane and residual neural tissue, leaving only a transparent retinal vascular network. The network was collected and moved to a glass slide, flattened, air-dried, and stained with PAS. Finally, changes in retinal capillaries in different groups were observed under a microscope.

### Terminal-deoxynucleotidyl transferase-mediated nick end labelling (TUNEL)

Paraffin sections were dewaxed with distilled water and then washed with PBS. Proteinase K working solution was added and incubated at 37 °C for 30 min. The sections were then washed with water and treated with H_2_O_2_ at room temperature for 10 min. After washing, TUNEL reaction solution was added and incubated at 37 °C for 60 min in the dark. The sections were washed again and then incubated with streptavidin-HRP solution for 30 min at 37 °C in the dark. DAB was used for colour development. Finally, the sections were counterstained with haematoxylin and processed for mounting, followed by panoramic scanning in bright field.

### Transmission electron microscopy

The sample was prefixed with 3% glutaraldehyde and then fixed with 1% osmium tetroxide. Dehydration was performed using a series of acetone solutions with increasing concentrations of 30% → 50% → 70% → 80% → 90% → 95% → 100% (with three changes of 100% concentration). The sample was then infiltrated and embedded in a mixture of dehydrating solution and Epon812 embedding resin at ratios of 3:1, 1:1, and 1:3, followed by embedding in Epon812. Thin sections of approximately 60–90 nm were prepared using an ultramicrotome, mounted on copper grids, and stained with uranyl acetate for 10–15 min and lead citrate for 1–2 min at room temperature. Finally, a JEM-1400FLASH transmission electron microscope produced by Japan Electronics was used to capture images of the copper mesh. Each copper mesh was first observed under 6000 times magnification, and images were captured of selected regions for detailed observation of specific abnormalities.

### Network pharmacological analysis

#### Identification of potential targets of JGSQ

*Rehmannia glutinosa* Libosch., *Dioscorea opposita* Thunb., *Cornus officinalis* Sieb. et Zucc., *Poria cocos* (Schw.) Wolf, *Paeonia suffruticosa* Andr., *Alisma orientalis* (Sam.) Juzep., *Aconitum carmichaelii* Debx. and *Cinnamomum cassia* Presl. were searched from the TCMSP database (https://old.tcmsp-e.com/tcmsp.php) and TCMIP database (http://www.tcmip.cn/TCMIP/index.php/Home/Index/All). From the TCMSP database, active ingredients with "OB ≥ 30%" and "DL ≥ 0.18" were filtered, and the corresponding target proteins were obtained. Duplicates of the targets of JGSQ obtained from the two databases were merged and removed to obtain potential targets of JGSQ.

#### Identification of potential targets of DR

Potential targets for DR were obtained by searching the “GeneCards”, “TTD”, and “DisGeNET” databases. After obtaining targets from the “GeneCards” database, those with relevance scores above the 75th percentile were selected. These targets were then merged with others from the other databases, and duplicates were removed to obtain the potential targets for DR.

#### Construction of the JGSQ-ingredient-DR intersection target network and protein‒protein interaction network

A Venn diagram (http://www.bioinformatics.com.cn/static/others/jvenn/index.html) was generated to identify the intersection between targets for JGSQ and DR targets, which may represent potential targets for the treatment of DR with JGSQ. The corresponding components and JGSQ for the intersection targets were screened, and a “JGSQ-component-target network” was constructed using Cytoscape 3.8.2. To investigate the protein‒protein interactions (PPIs) of JGSQ in treating DR, the drug-intersecting genes were uploaded to the STRING interaction database (https://string-db.org/) for PPI network construction. The species was set as “Homo sapiens”, and the minimum interaction threshold was set as “highest confidence” > 0.4, while other parameters were set at the default values. The resulting data were imported into Cytoscape 3.8.2 for network analysis, and core targets were selected to generate the PPI network.

#### GO and KEGG enrichment analysis

The function of intersecting targets was annotated using the online tool DAVID (https://david.ncifcrf.gov/), the “select identifier” was set to “official gene symbol”, the “list type” was set to “genelist”, and “homo sapiens” was chosen as the species. GO analysis was performed separately for biological processes, cellular components, and molecular functions, as well as KEGG analysis. Save the enrichment results as a table or diagram.

#### Molecular docking

AutoDock Vina (1.1.2) was used to perform molecular docking of drug active ingredients and key target proteins to validate their interaction activity. (1) Compounds were downloaded in mol2 format from the TCMSP official website and then imported into Chembio3D for energy minimization. Afterwards, it was imported into AutodockTools-1.5.6 for hydrogenation, charge calculation, and assignment. (2) The target protein was downloaded from the Protein Data Bank (http://www.rcsb.org/). (3) The protein was imported into PyMOL (2.3.0) to remove the original ligand and water molecules and then imported into AutodockTools (v1.5.6) for hydrogenation, charge calculation, charge assignment, and atom type specification. (4) Use the original protein ligand as the docking box centre. If there was no original ligand, the region near the reported key amino acid residues was used as the docking area. (5) The interaction mode was analysed using PyMOL and Ligplot.

### Immunofluorescence

After fixing the eyeball tissue specimens in 4% paraformaldehyde solution for 48 h, they were processed into paraffin-embedded sections. The paraffin sections were subjected to dewaxing and gradient alcohol dehydration, followed by antigen retrieval. After washing, the sections were blocked with serum for 30 min, incubated with antibodies, and finally stained and observed under a fluorescence microscope. The image was collected using CaseViewer.

#### Preparation of JGSQ-containing serum

JGSQ-containing serum was prepared according to a previously published study [17]. SD rats (200 ± 10 g, Male) purchased from Jiangsu GemPharmatech Co., Ltd were split into two groups after 7 days of acclimation. JGSQ-containing serum group was treated with JGSQ (2 g/mL, 1 mL/100 g) daily for 7 days by oral gavage and the normal serum group was given water. Two hours after the final administration, the rats were anesthetized with pentobarbital (50 mg/kg), the serum was collected and filtered through 0.22 µm strainers.

#### Cell lines and regents

Mouse retinal ganglion cells (RGC-5) were purchased from the BeNa Culture Collection (cat: 23082201, Beijing, China). Cells were cultured in DMEM (cat: C11995500BT, Gibco, China) containing 10% foetal bovine serum (cat: 10099-141C, Gibco, USA) and 1% penicillin/streptomycin (cat: 15140148, Gibco, USA) and placed at 37 °C in a humidified incubator containing 5% CO_2_. After the cells grew to the logarithmic growth phase, they were digested with 0.25% trypsin–EDTA 1X (cat: 25200-072, Gibco, USA) and passaged 1:3. The AKT activator SC97 (cat: HY-18749) and HIF-1α activator fenbendazole-d3 (cat: HY-B0413S) were purchased from MedChemExpress (New Jersey, USA).

#### Western blot

RIPA containing protease inhibitors was added to the processed retinal tissues and kept on ice for 30 min. After complete lysis, the cell suspension was centrifuged at 10,000 rpm for 5 min at 4 °C. The protein concentration of the supernatant was then measured using the BCA protein assay kit. The protein of each sample was separated by 10% SDS‒PAGE and transferred onto a PVDF membrane. Seal with a solution containing 5% skim milk for 1 h. Then, the cells were incubated with the following antibodies: TP53 (cat: 60283-2-Ig, 1:5000, Proteintech), AKT (cat: 60203-2-Ig, 1:5000, Proteintech), p-AKT (cat: 66444-1-Ig, 1:5000, Proteintech), HIF-1α (cat: AF7087, 1:5000, Beyotime), cleaved PARP (cat: AF1567, 1:1000, Beyotime), GAPDH (cat: 60004-1-Ig, 1:5000, Proteintech), and β-actin (cat: 48139 1:5000, SAB Antibody) overnight at 4 °C. Subsequently, the membranes were washed with TBST and incubated with goat anti-rabbit/mouse IgG (H + L) HRP at room temperature for 1 h. The membrane was exposed with chemiluminescence detection reagent. ImageJ 6.0 software was used to obtain the band intensity of protein expression and perform analysis, normalized to the band intensity of β-actin. All experiments were repeated three times.

#### Statistic analysis

Statistical analysis was performed using SPSS 25.0 and GraphPad Prism (Version: 6.02, USA) software. Data are presented as the mean ± standard deviation. Differences between groups were analysed using one-way analysis of variance (ANOVA) and two-tailed Student's t test. A p value < 0.05 was considered statistically significant for all comparisons.

## Results

### JGSQ alleviates retinal lesions in *db/db* mice with DR

**Fig. 1:**
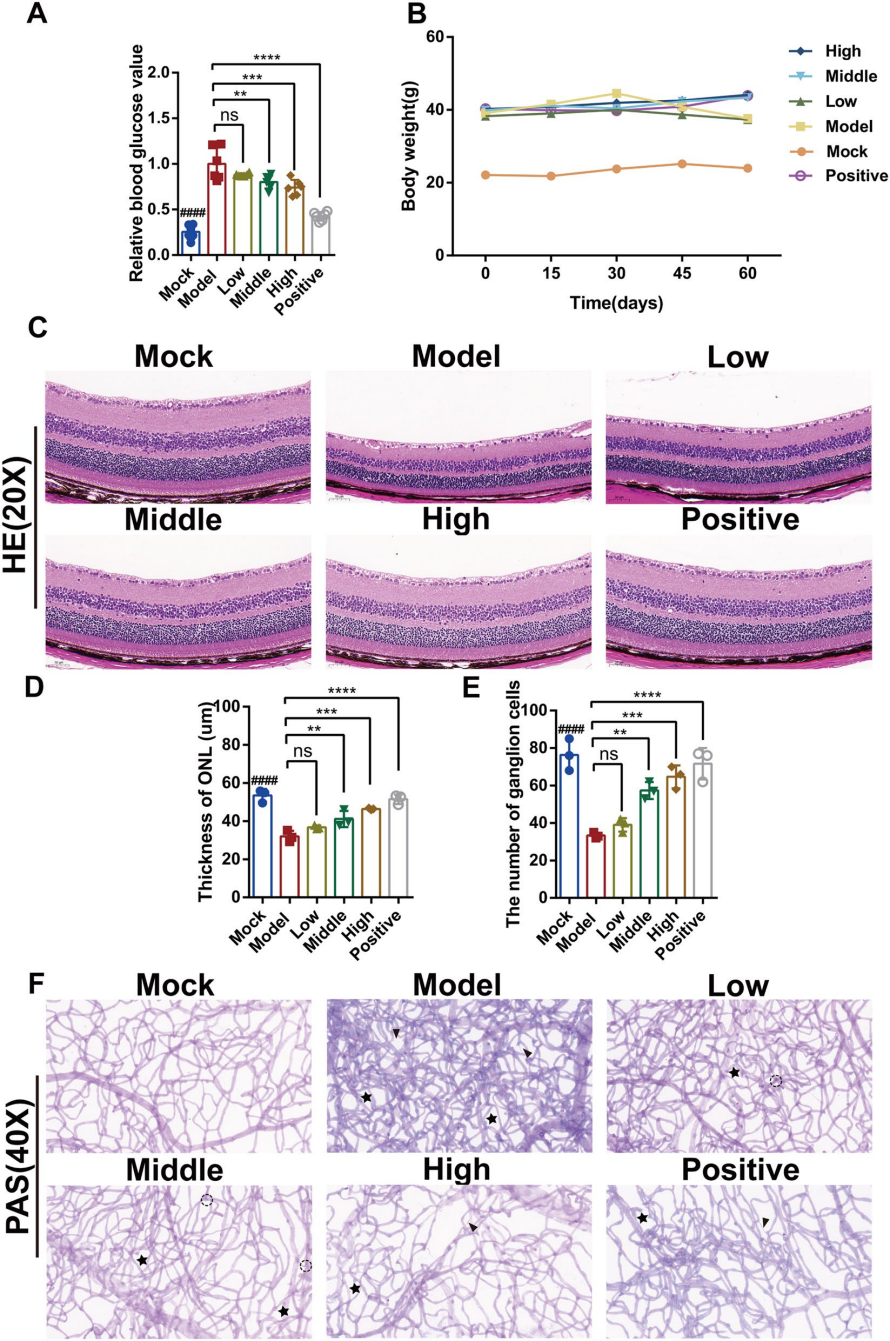
JGSQ improves retinal lesions in *db/db* mice with DR. **A** The relative blood glucose value of *db/db* and *db/m* mice. **B** The body weight of *db/db* and *db/m* mice, weighed once every 2 weeks. **C** Images of H&E staining of retina from mice (magnification × 20). The image was captured by CaseViewer. **D** The thickness of outer nuclear layer (ONL) were measured by Adobe Illustrate. **E** The number of ganglion cells were calculated by Image J. **F** PAS staining of retinal vascular network patches (magnification × 40) were used to identify the abnormal lesions of retinal blood vessels. Mock VS Model. ^ns^*P* > 0.05, ^#^*P* < 0.05, ^##^*P* < 0.01, ^###^*P* < 0.001, ^####^*P* < 0.0001. JGSQ-treated VS Model, Positive VS Model. ^ns^*P* > 0.05, *P < 0.05, **P < 0.01, ***P < 0.001, ****P < 0.0001

After 8 weeks of gavage, *db/db* mice reached 18 weeks of age and met the modelling criteria for DR [18]. Blood glucose testing revealed that JGSQ improved blood glucose levels in *db/db* mice, although its hypoglycaemic effects were not as strong as those of metformin (Fig. 1A). Meanwhile, the model group mice showed rapid weight gain but experienced significant weight loss after one month, and JGSQ and metformin maintained stable body weight (Fig. 1B). Based on the H&E staining results (Fig. 1C), it is apparent that the JGSQ pill can improve the thickness of the outer nuclear layer of the retina in a dose-dependent manner (Fig. 1D) and inhibit the quantity of apoptosis in retinal ganglion cells (Fig. 1E). Furthermore, the results from PAS staining of the retinal vascular network demonstrated that the model group exhibited significant microaneurysms and acellular capillaries when compared to the control group, and both JGSQ and metformin improved abnormal vascular morphology to a certain extent (Fig. 1F). However, the improvement with metformin was better than that with JGSQ.

### JGSQ reduces retinal ganglion cell apoptosis in *db/db* mice with DR

**Fig. 2:**
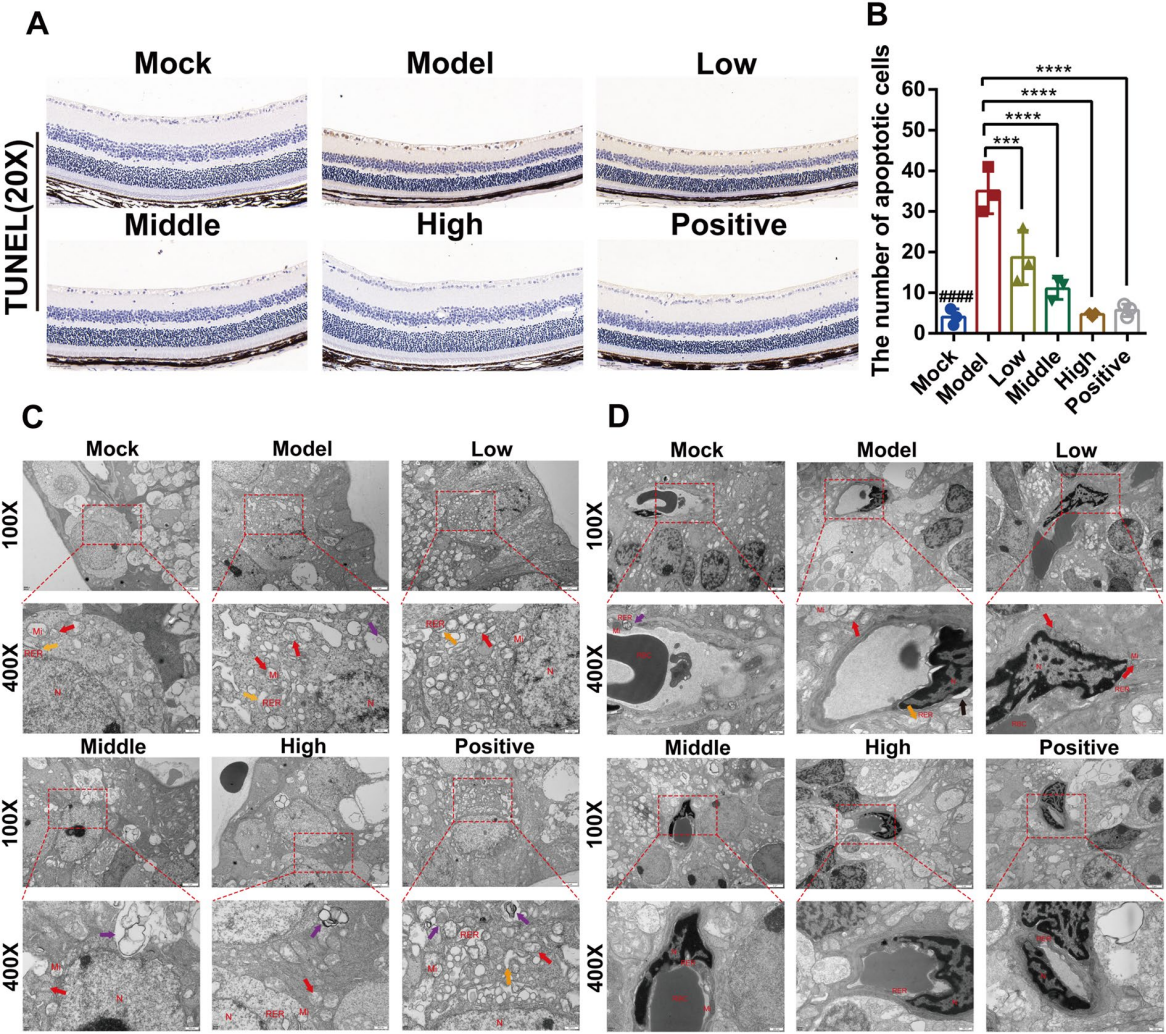
JGSQ alleviates retinal ganglion cell apoptosis in *db/db* mice with DR. **A** TUNEL staining (magnification × 20) was used to identify the cell apoptosis in retina. **B** The number of apoptotic cells were analyzed using GraphPad Prism. **C**, **D** The morphology and structure of ganglion cells and endothelial cells were observed by transmission electron microscopy. Cell nucleus (N), mitochondria (Mi), rough endoplasmic reticulum (RER), Red blood cell (RBC), mitochondrial swelling (), rough endoplasmic reticulum dilatation (), autophagy (). **D** The morphology and structure of vascular endothelial cells were observed by transmission electron microscopy. Mock VS Model. ^ns^*P* > 0.05, ^#^*P* < 0.05, ^##^*P* < 0.01, ^###^*P* < 0.001, ^####^*P* < 0.0001. JGSQ-treated VS Model, Positive VS Model. ^ns^*P* > 0.05, *P < 0.05, **P < 0.01, ***P < 0.001, ****P < 0.0001

The TUNEL staining results showed that significant apoptosis of retinal ganglion cells occurred in the model group. However, JGSQ and metformin significantly improved retinal ganglion cell apoptosis. Moreover, high-dose JGSQ had a better inhibitory effect on retinal ganglion cell apoptosis than metformin (Fig. 2A, B). Furthermore, this study found that the neural cells in the model group exhibited significant abnormalities in their morphological structure through transmission electron microscopy analysis, with swollen mitochondria, shortened or fragmented cristae, reduced granules in the matrix, significant expansion of the rough endoplasmic reticulum, widened intermembrane space of the vesicular structure, and visible autophagy in the cytoplasm. JGSQ exhibited a significant dose-dependent improvement in neural cell morphology, alleviating mitochondrial swelling and autophagy, while the effect of metformin was not as significant as that of JGSQ (Fig. 2C). Moreover, the results of retinal vascular endothelial cells showed that the model group also experienced abnormal morphological structures, irregular cell nuclei, heterochromatic edges, and vacuoles around the nucleus. Both JGSQ and metformin improved this phenotype, but the effect of metformin was better. The endothelial structure of the positive group was the same as that of the control group, which was of normal morphology (Fig. 2D). From the above phenotypic results, it can be inferred that JGSQ has a better therapeutic effect on improving retinal ganglion cell apoptosis but is inferior to metformin in terms of reducing blood sugar levels and improving vascular neogenesis or abnormal vascular morphology.

### Network pharmacology analysis of JGSQ in the treatment of DR: identification of the main molecular targets and signalling pathways

**Fig. 3:**
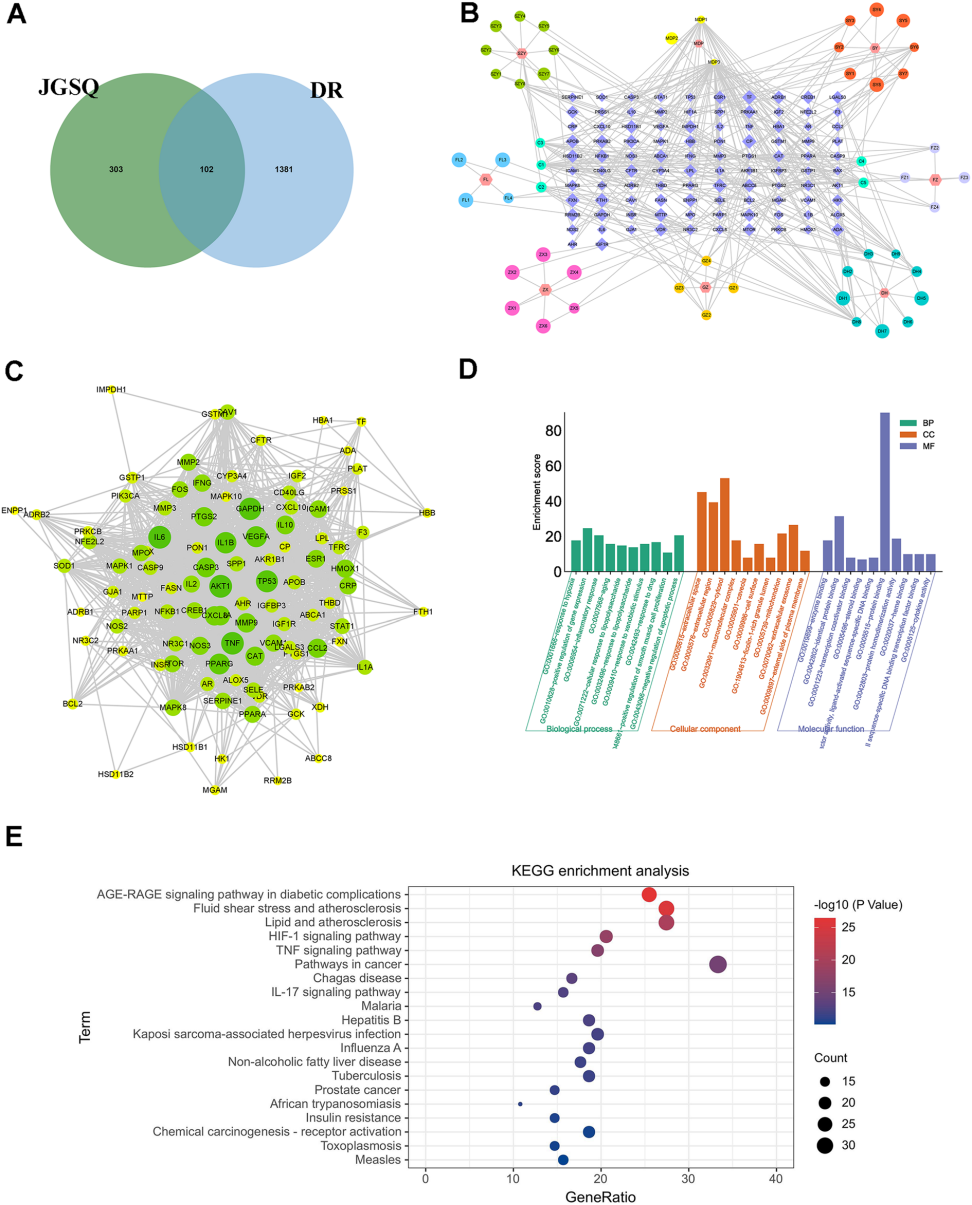
Network pharmacology analysis of JGSQ in the treatment of DR. **A** A venn diagram between targets for JGSQ and DR targets, which may represent potential targets for the treatment of DR with JGSQ; **B** JGSQ-Ingredient-DR Intersection Target Network, core nodes of the network were identified as targets with high Betweenness Centrality, Closeness Centrality, and Degree greater than the median. **C** PPI network of JGSQ in treating DR, the size and color of nodes reflect the size of the Degree, with larger nodes indicating higher Degree values. The thickness of edges is used to reflect the size of the Combine score, with thicker edges indicating higher Combine scores. This graph is used to select core targets for constructing a PPI network; **D** GO enrichment analysis; **E** KEGG enrichment analysis

In this study, 406 potential targets for JGSQ were identified through the retrieval of active ingredients from the TCMSP database, and 113 compounds of JGSQ were identified including 21 from *Aconitum carmichaelii* Debx., 7 from *Cinnamomum cassia* Presl, 13 from *Rehmannia glutinosa* Libosch., 16 from *Dioscorea opposita* Thunb., 20 from *Cornus officinalis* Sieb. et Zucc., 15 from *Poria cocos* (Schw.) Wolf, 11 from *Paeonia suffruticosa* Andr., and 10 from *Alisma orientalis* (Sam.) Juzep. A total of 1483 relevant targets for diabetic retinopathy were identified, and 102 of these targets were found to intersect with potential targets of JGSQ, as shown in Fig. 3A.

Subsequently, these potential targets were constructed into a target network forming the intersection graph of DR and JGSQ (Fig. 3B). The PPI network of the intersection of disease and drug targets was constructed using the STRING platform (Fig. 3C). After screening based on network topology properties, the top ten nodes in terms of degree centrality in the network were IL6, AKT1, TNF, GAPDH, TP53, IL1B, VEGFA, PPARG, CASP3, and PTGS2. These nodes mainly regulate cell apoptosis and inflammatory cytokines. Moreover, this study also conducted GO (Fig. 3D) and KEGG enrichment analyses (Fig. 3E). We found that the AGE-RAGE signalling pathway and HIF-1 signalling pathway may be the main signalling pathways through which JGSQ improves DR.

### Identification of the main active ingredients of JGSQ and docking targets at the molecular level

**Fig. 4:**
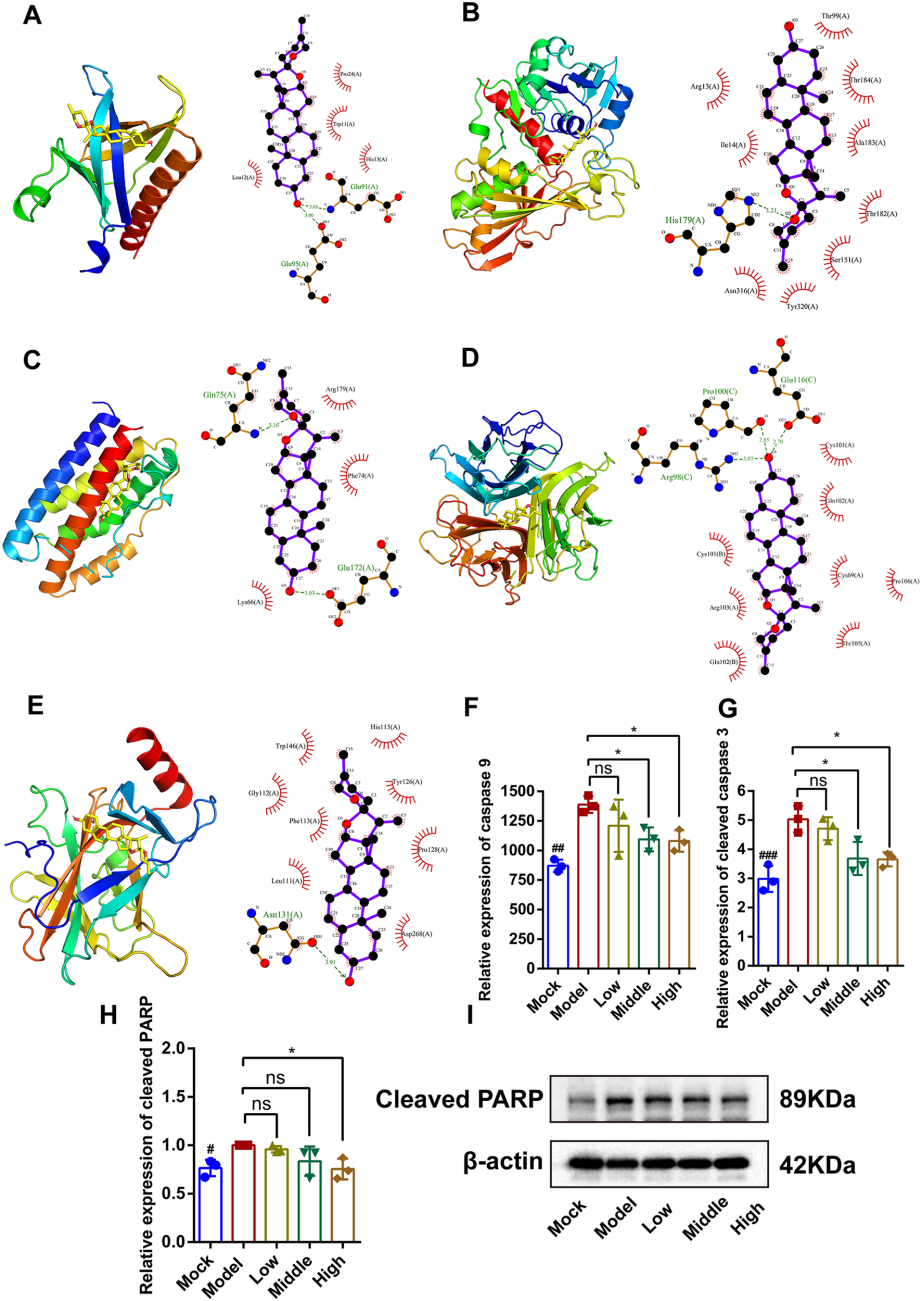
Molecular docking. Diosgenin is the main active compound that can bind with key targets. **A** Diosgenin binds AKT1; **B** Diosgenin binds GAPDH; **C** Diosgenin binds IL6; **D** Diosgenin binds TNF; **E** Diosgenin binds TP53. **F**, **G** The expression levels of cleaved caspase 3 and caspase 9 in retinal tissue using ELISA. **H**, **I** Western blot detected the expression of cleaved PARP in *db/db* mice retina tissue

In addition, this study conducted semiflexible docking of the top five important targets and compounds with high degree values based on network pharmacology analysis. Affinity is used to indicate how well a small molecule binds to its target protein. A binding energy less than 0 means that a small molecule can bind to its target protein freely, and the smaller the value is, the higher the possibility of binding **(**Table 2**)**. Interestingly, this study found that diosgenin is the main active ingredient that can bind with key targets. Diosgenin forms hydrogen bonds with Glu91 and Glu95 of AKT1, with hydrogen bond lengths of 3.03 Å and 3.00 Å, respectively (Fig. 4A). Diosgenin forms hydrogen bonds with His179 of GAPDH, and the length of the hydrogen bond is 3.21 Å (Fig. 4B). Diosgenin also forms hydrogen bonds with Glu172 and Gln75 of IL6, and the lengths of the hydrogen bonds are 3.03 Å and 3.16 Å, respectively (Fig. 4C). Diosgenin forms hydrogen bonds with Glu116, Pro100, and AArg98 of TNF with bond lengths of 2.70 Å, 2.85 Å, and 3.07 Å, respectively (Fig. 4D); diosgenin also forms a hydrogen bond with Asn131 of TP53 with a bond length of 3.91 Å (Fig. 4E). Additionally, the UHPLC‒QE‒MS results from JGSQ demonstrated that diosgenin is one of the main components of JGSQ. Therefore, it can be inferred that diosgenin may be the main active ingredient in JGSQ, and AKT, GAPDH, TNF, TP53, and IL-6 may be the main targets of JGSQ.

**Table 2:** Docking results between core small molecules and core target proteins

Targets	PDB ID	Compounds	Affinity(kcal/mol)	Targets	PDB ID	Compounds	Affinity(kcal/mol)
IL6	1ALU	Beta-sitostero	− 6.8	TNF	2E7A	Beta-sitostero	− 8.3
		Sitosterol	− 6.7			Sitosterol	− 8.9
		Stigmasterol	− 6.8			Stigmasterol	− 8.8
		( +)-catechin	− 6.2			( +)-catechin	− 9.1
		Diosgenin	− 7.6			Diosgenin	− 10.8
GAPDH	6YND	Beta-sitostero	− 7.5	AKT1	1UNQ	Beta-sitostero	− 7.2
		Sitosterol	− 7.3			Sitosterol	− 7.2
		Stigmasterol	− 7.5			Stigmasterol	− 7.1
		( +)-catechin	− 7			( +)-catechin	− 5.9
		Diosgenin	− 8.6			Diosgenin	− 8.1
TP53	1UOL	Beta-sitostero	− 6.6				
		Sitosterol	− 6.4				
		Stigmasterol	− 6.8				
		( +)-catechin	− 6.1				
		Diosgenin	− 7.7

### JGSQ reduces the expression of DR inflammatory factors and regulates TP53 to improve cell apoptosis

In previous studies, this study found that the main pharmacological effect of JGSQ on DR is to protect retinal ganglion cells from apoptosis. To further investigate the apoptotic inhibition of retinal ganglion cells by JGSQ, we examined the expression levels of cleaved caspase 3 and caspase 9 in retinal tissue using ELISA. The results, as shown in Fig. 4F, G, indicated a reduction in cleaved caspase 3 and caspase 9 expression in the JGSQ-treated groups. Western blot results showed that JGSQ decreased the expression of cleaved PARP in the retinal tissue of *db/db* mice ( Fig. 4H, I).

**Fig. 5:**
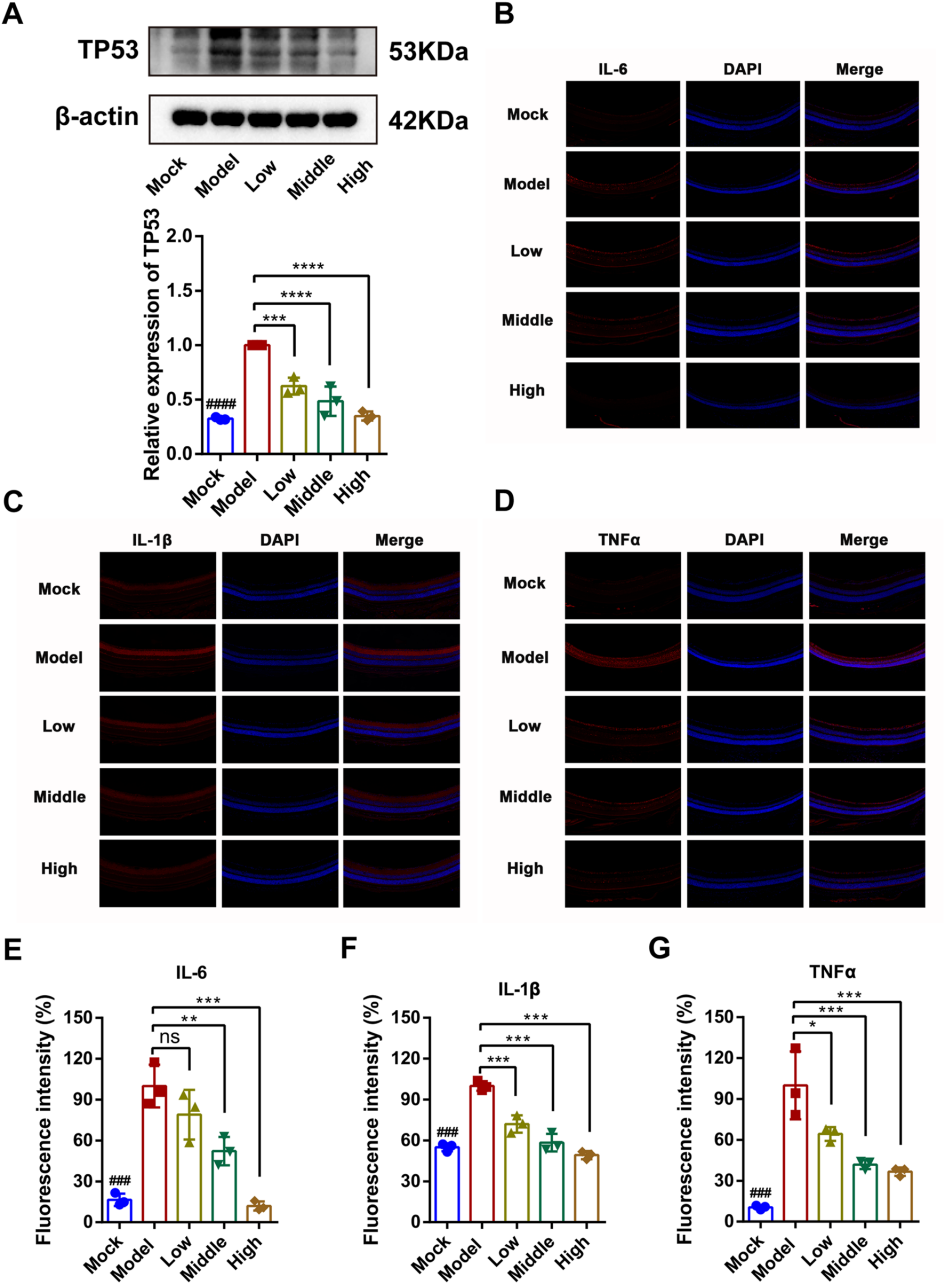
JGSQ reduces the expression of DR inflammatory factors and regulates TP53 to improve cell apoptosis. **A** Western blot was used to detect the effect of JGSQ on TP53 proteins, quantified by ImageJ software. **B**–**D** The expressions of IL-6, IL-1β and TNFα were detected by immunofluorescence. **E**–**G** Statistical analyses of IL-6, IL-1β and TNFα were performed using GraphPad Prism. Mock VS Model. ^ns^*P* > 0.05, ^#^*P* < 0.05, ^##^*P* < 0.01, ^###^*P* < 0.001, ^####^*P* < 0.0001. JGSQ-treated VS Model. ^ns^*P* > 0.05, *P < 0.05, **P < 0.01, ***P < 0.001, ****P < 0.0001

Subsequently, based on the results of network pharmacological analysis, the TP53 protein is a classic transcription factor that regulates the cell cycle and apoptosis[19], and considering that AKT is an upstream target of TP53[20], it can be inferred that JGSQ may regulate TP53 to protect retinal ganglion cells from apoptosis by promoting AKT phosphorylation. Additionally, JGSQ may improve the inflammatory state of DR by improving the levels of inflammatory factors such as TNFα and IL-6. Therefore, this study detected the expression of TP53 by western blotting and found that JGSQ significantly inhibited the expression of TP53 (Fig. 5A). In addition, immunofluorescence staining of TNFα, IL-6, and IL-1β revealed that JGSQ also significantly inhibited the expression of TNFα, IL-6, and IL-1β (Fig. 5B–G).

### JGSQ mainly improves DR through the Akt/HIF-1α pathway

**Fig. 6:**
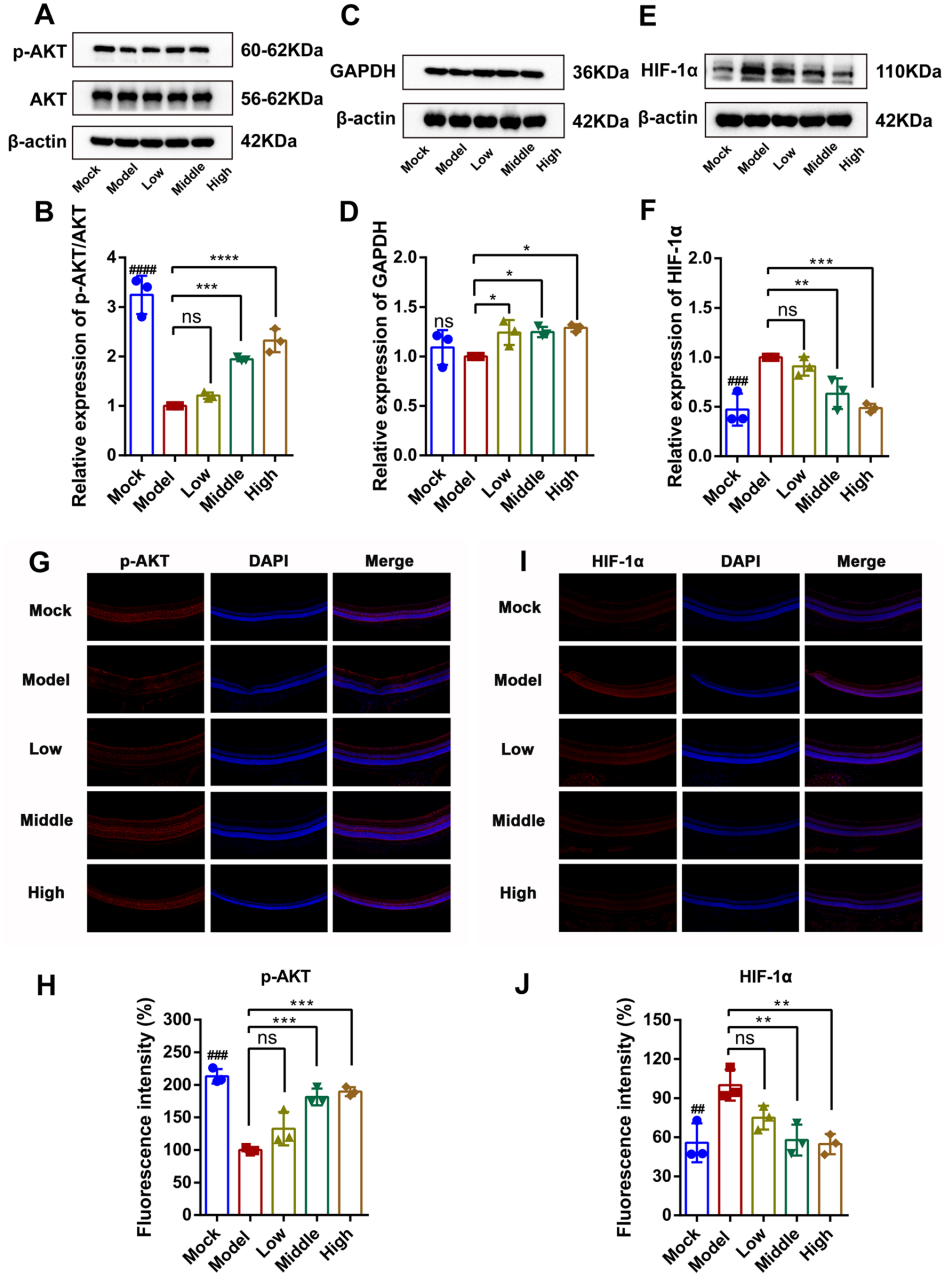
JGSQ mainly improves DR through the Akt/HIF-1α pathway. **A**-**F** Western blot was used to detect the effect of JGSQ on AKT, p-AKT, GAPDH, HIF-1α proteins, quantified by ImageJ software. Statistical analyses of IL-6, IL-1β and TNFα were performed using GraphPad Prism. **G**–**I** The expressions of p-AKT and HIF-1α were detected by immunofluorescence. (H–J) Statistical analyses of p-AKT and HIF-1α were performed using GraphPad Prism. Mock VS Model. ^ns^*P* > 0.05, ^#^*P* < 0.05, ^##^*P* < 0.01, ^###^*P* < 0.001, ^####^*P* < 0.0001. JGSQ-treated VS Model. ^ns^*P* > 0.05, *P < 0.05, **P < 0.01, ***P < 0.001, ****P < 0.0001

Due to the ability of JGSQ to suppress the expression of TP53 and inflammatory factors, we conducted western blot analysis on the upstream target AKT and found that JGSQ could promote the expression of p-AKT (Fig. 6A, B). In addition, this study also verified the expression of GAPDH via western blot analysis and found that JGSQ had no effect on GAPDH (Fig. 6C, D). Based on the previous analysis of network pharmacology, the HIF-1 signalling pathway may be the main signalling pathway by which JGSQ improves DR. One of the main reasons for the induction of retinal ganglion cell apoptosis is the combination of hypoxia and ischaemia [10, 11]. HIF-1α is a major target that regulates hypoxia, cell apoptosis and inflammation, and AKT can mediate the activation of HIF-1α to induce the progression of DR [21, 22]. Therefore, western blot analysis was performed to study the effect of JGSQ on HIF-1α, which was found to be significantly inhibited (Fig. 6E, F). To further validate that JGSQ mainly improved DR by protecting retinal ganglion cells from apoptosis through the Akt/HIF-1α pathway, immunofluorescence staining was conducted for p-AKT (Fig. 6G, H) and HIF-1α (Fig. 6I, J). The staining results showed that JGSQ could improve the pathological changes of DR by regulating the Akt/HIF-1α pathway**.**

For further mechanistic investigation, we selected RGC-5 cells for in vitro experiments. Therefore, we used serum containing JGSQ, the AKT activator SC79 and the HIF-1α activator fenbendazole-d3 to observe the effects of JGSQ on TP53 and apoptosis-related proteins through regulation of the AKT/HIF-1α pathway. Firstly, based on the results of the CCK-8 assay, we determined that a glucose concentration of 50 mM could be used to establish a mouse retinal ganglion cell injury model (Additional file 2: Fig. 2A). The results demonstrate that the containing serum can promote AKT, inhibit HIF-1α, and reduce the expression of TP53 and cleaved PARP (Additional file 2: Fig. 2B, C). The ELISA results revealed that the levels of cleaved caspase 3 and caspase 9 in lysed RGC-5 cells were suppressed by JGSQ (Additional file 2: Fig. 2D, E). Moreover, the effect of JGSQ was attenuated by the activation of AKT and HIF-1α, suggesting that JGSQ exerts its action partly through these targets.

## Discussion

DR has always been the most common microvascular complication of diabetes and remains a leading cause of blindness [23]. Studies have shown that sustained high blood glucose in diabetes can lead to excessive activation and proliferation of microglia, which produce various proinflammatory and cytotoxic factors, leading to chronic inflammation and causing damage to the retinal neurovascular unit, blood‒retinal barrier breakdown, and further deterioration of DR [24]. Inhibiting vascular endothelial damage and protecting against retinal ganglion cell apoptosis are some of the main ways to prevent the occurrence of DR. Neuroprotection as a new approach for the treatment of the early stages of DR has been particularly emphasized [25].

JGSQ is a traditional Chinese herbal formula that contains 8 herbs and has been used for thousands of years in China. This traditional Chinese herbal formula was historically used to treat blurred vision in ancient China. In modern research, it has been discovered that it can improve diabetes [15]. Interestingly, according to the results of UHPLC‒QE‒MS analysis in the Supplementary Figure, this study found that many components in JGSQ have been proven to effectively improve DR. These components include 6-shogaol [26], emodin [27], kaempferol [28], quercetin [29, 30], geniposide [31], gallic acid [32], paeoniflorin [33], formononetin [34], wogonin [35], and asiatic acid [36]. Therefore, it is reasonable to believe that JGSQ has the potential to improve DR. According to previous literature research, 18-week-old *db/db* mice have been successfully used as mouse models for DR [18]. In this study, *db/db* mice with DR were treated with JGSQ orally. We euthanized the mice when they reached 18–19 weeks of age and confirmed the presence of DR characteristics through H&E staining. The blood glucose levels and body weights of the mice were monitored, and the results showed that JGSQ reduced blood glucose levels to a certain extent, but the effect was not as significant as that of metformin. Subsequently, it was demonstrated through H&E and TUNEL staining that JGSQ can effectively reduce the apoptosis of retinal ganglion cells with DR. Transmission electron microscopy results also showed the alleviation of apoptosis in retinal ganglion cells. Therefore, we have reasonable evidence that JGSQ primarily improves DR by protecting retinal ganglion cells from apoptosis. However, it does not have a significant advantage over metformin in improving the structure of vascular endothelial cells.

To further understand the mechanism of how JGSQ alleviates DR, this study conducted a network pharmacology analysis and found that JGSQ may primarily act on IL6, AKT1, TNF, GAPDH, TP53, IL1B, VEGFA, PPARG, CASP3, and PTGS2 as target proteins. These targets suggest that JGSQ mainly regulates the chronic inflammatory microenvironment and cell proliferation and apoptosis related to DR. IL-6, IL-1β, and TNFα are classic proinflammatory cytokines, while PTGS2 is the gene name for COX-2, which regulates the inflammation of DR [37]. On the other hand, AKT1, TP53, and CASP3 targets are closely related to cell proliferation and apoptosis [19, 38]. The findings were consistent with the results from phenotypic analysis, including H&E and TUNEL staining and transmission electron microscopy, which confirmed that JGSQ mainly improves DR by effectively inhibiting the apoptosis of retinal ganglion cells and inflammation.

To further clarify the active ingredients and core targets of JGSQ, this study performed molecular docking of the top five core ingredients of JGSQ with the top ten core targets. Surprisingly, it was found that diosgenin can perfectly bind to AKT, GAPDH, TNF, TP53, and IL-6 simultaneously based on the scoring results of molecular docking. Additionally, data from UPLC‒MS also confirm that diosgenin is also one of the main active compounds in JGSQ. Therefore, it can be reasonably believed that diosgenin is the core ingredient of JGSQ. Many studies have also proven that diosgenin can improve many complications of diabetes, such as diabetic nephropathy [39], nonalcoholic fatty liver disease in type 2 diabetes [40], cognitive impairment [41], as well as DR [42, 43] Therefore, this study used AKT, GAPDH, TNF, TP53, and IL-6 as the core targets of JGSQ's action, and the above five targets are the main molecules that regulate cell proliferation and inflammation. Subsequently, this study further validated the expression of cleaved PARP and TP53 through western blotting and found that JGSQ can downregulate the expression of cleaved PARP and TP53. Additionally, this study validated the expression of cleaved caspase 3 and caspase 9 by ELISA and the results showed JGSQ can also reduce the expression of cleaved caspase 3 and caspase 9. The above experiment demonstrates that JGSQ can inhibit the apoptosis of retinal ganglion cells induced by diabetes. In addition, retinal immunofluorescence staining was performed to analyse the expression of IL6, TNFα and IL-1β in the retina, and JGSQ effectively inhibited the expression of proinflammatory cytokines. AKT is a known upstream target of TP53 in the PI3K-AKT signalling pathway, and this study further found that JGSQ can promote the expression of p-AKT. At this point, we can conclude that promoting the expression of AKT and inhibiting the expression of TP53 by JGSQ can suppress cell apoptosis and improve the phenomenon of photoreceptor cell apoptosis.

**Fig. 7:**
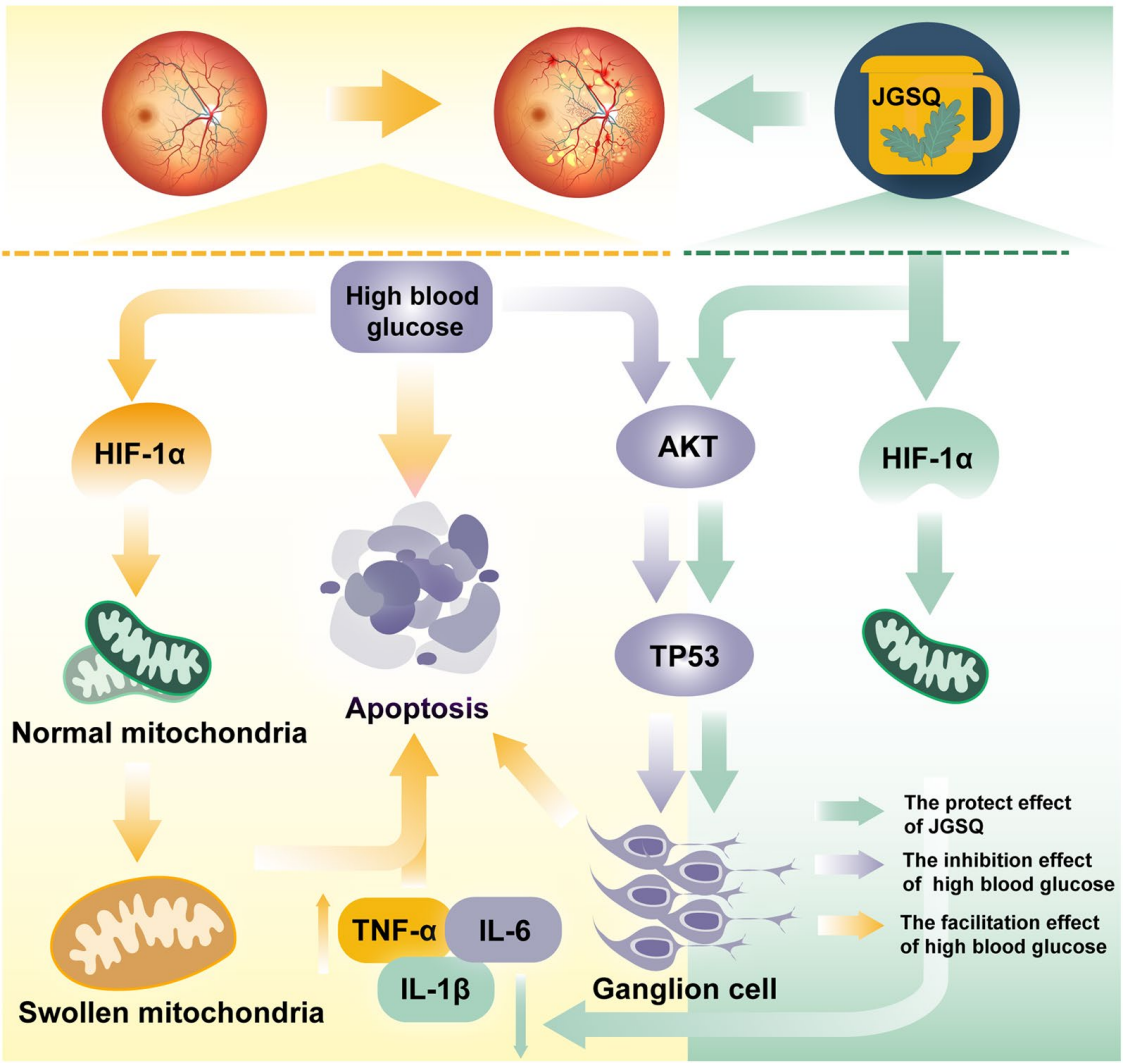
Mechanism of JGSQ intervention in diabetic retinopathy

Similarly, anti-inflammatory agents and protection against neuronal cell apoptosis are the main treatment methods for DR, and JGSQ can inhibit the release of inflammatory cytokines [4, 7, 44]. It can be concluded that JGSQ primarily protects retinal ganglion cells from apoptosis by upregulating p-AKT and suppressing the expression of inflammatory cytokines, thereby improving DR. However, the previous pharmacological network analysis also revealed that the AGE-RAGE signalling pathway and HIF-1 signalling pathway were the most relevant signalling pathways regulated by JGSQ for improving DR, as determined by KEGG enrichment analysis. Ischaemia and hypoxia are the main causes of retinal ganglion cell apoptosis, and the HIF-1 signalling pathway plays a critical role in this process [10]. Moreover, studies have shown that AKT is involved in the process of hypoxia**,** HIF-1α can also regulate AKT [45], and AKT can also mediate the activity and expression of HIF-1α [21, 22, 46, 47]**.** Furthermore, HIF-1α can also regulate the expression of inflammatory cytokines in cells [48, 49]. When diabetic retinopathy develops, the microenvironment in the retina experiences hypoxia, which triggers an increase in the expression of HIF1α. Consequently, the inhibition of AKT enhances the expression of p53, resulting in the apoptosis of RGCs. AKT further activates the expression of HIF-1α. Akt increases the stability of HIF-1α by phosphorylating and inhibiting its degradation mechanism. Additionally, HIF-1α can stimulate the release of inflammatory cytokines, aggravating the apoptosis of retinal ganglion cells and thereby exacerbating the process of DR. Therefore, AKT and HIF-1α may be the core therapeutic targets of JGSQ. Therefore, this study further explored the upstream targets of JGSQ through western blotting and immunofluorescence and found that JGSQ can inhibit the expression of HIF-1α and promote p-AKT. It can be inferred that JGSQ can mainly protect retinal ganglion cells from apoptosis and improve the inflammatory environment of DR through the Akt/HIF-1α pathway. In vitro experimental results confirmed that both AKT and HIF-1α act as key targets in retinal cell apoptosis, and the effect of JGSQ was attenuated by the activation of AKT and HIF-1α, suggesting that JGSQ exerts its action partly through AKT/HIF-1α (Fig. 7).

## Conclusion

**Fig. 8:**
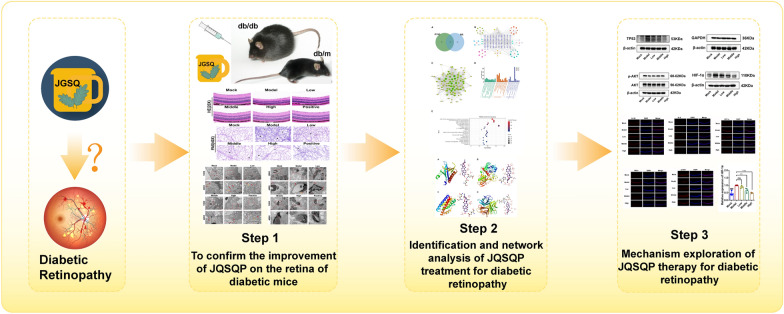
Graphical Abstract

This study further confirms and explores the effective basis of JGSQ in improving diabetic retinopathy, indicating that JGSQ can protect retinal ganglion cells from apoptosis. In *vitro* experiments validated the results of network pharmacology and molecular docking, showing that diosgenin is the core active ingredient of JGSQ in treating DR, and JGSQ improves DR by regulating the Akt/HIF-1α pathway. Before the effective monomeric molecules of JGSQ compounds enter clinical trials, further investigations are needed. This provides a reference for further preclinical studies (Fig. 8).

### Supplementary Information

**Additional file 1: Figure S1.** UHPLC-QE-MS of JGSQ.

**Additional file 2: Figure S2.** The target of JGSQ was validated in vitro. (A) Cell survival rate of RGC-5 cells under different concentrations of glucose. (B) The containing serum of JGSQ combined with the activators of AKT and HIF-1α respectively to alleviated the apoptosis of RGC-5 cells. (C) Statistical analyses of TP53 and cleaved PARP were performed using GraphPad Prism. (D-E) The levels of cleaved caspase 3 and caspase 9 in lysed RGC-5 cells were detected by ELISA kits. Groups VS Model.^ns^*P* >0.05, *P < 0.05, **P < 0.01, ***P < 0.001, ****P < 0.0001. AKT activator: SC79; HIF-1α activator: fen-d3 (fenbendazole-d3).

## Data Availability

Data will be made available on request.

## Abbreviations

DR: Diabetic Retinopathy
JGSQ: Jin-Gui-Shen-Qi Wan
T2DM: Type 2 diabetes mellitus
ANOVA: Analysis of variance
GO: Gene Ontology
H&E: Hematoxylin and eosin
KEGG: Kyoto Encyclopedia of Genes and Genomes
TUNEL: Terminal -deoxynucleotidyl transferase mediated nick end labeling
IF: Immunofluorescence
AKT: Protein kinase B
PBS: Phosphate buffer saline
PVDF: Polyvinylidene difluoride
HIF-1α: Hypoxia inducible factor-1 alpha
IL-6: Interleukin-6
TNFα: Tumor necrosis factor alpha
IL-1β: Interleukin-1 beta
SDS-PAGE: Sodium dodecyl sulfate polyacrylamide gel electrophoresis
