# Supplementary figures and images for: Jin-Gui-Shen-Qi Wan ameliorates diabetic retinopathy by inhibiting apoptosis of retinal ganglion cells through the Akt/HIF-1α pathway
Source: Chin Med. 2023 Oct 12;18:130. doi: 10.1186/s13020-023-00840-7 (PMC10568827; doi:10.1186/s13020-023-00840-7)

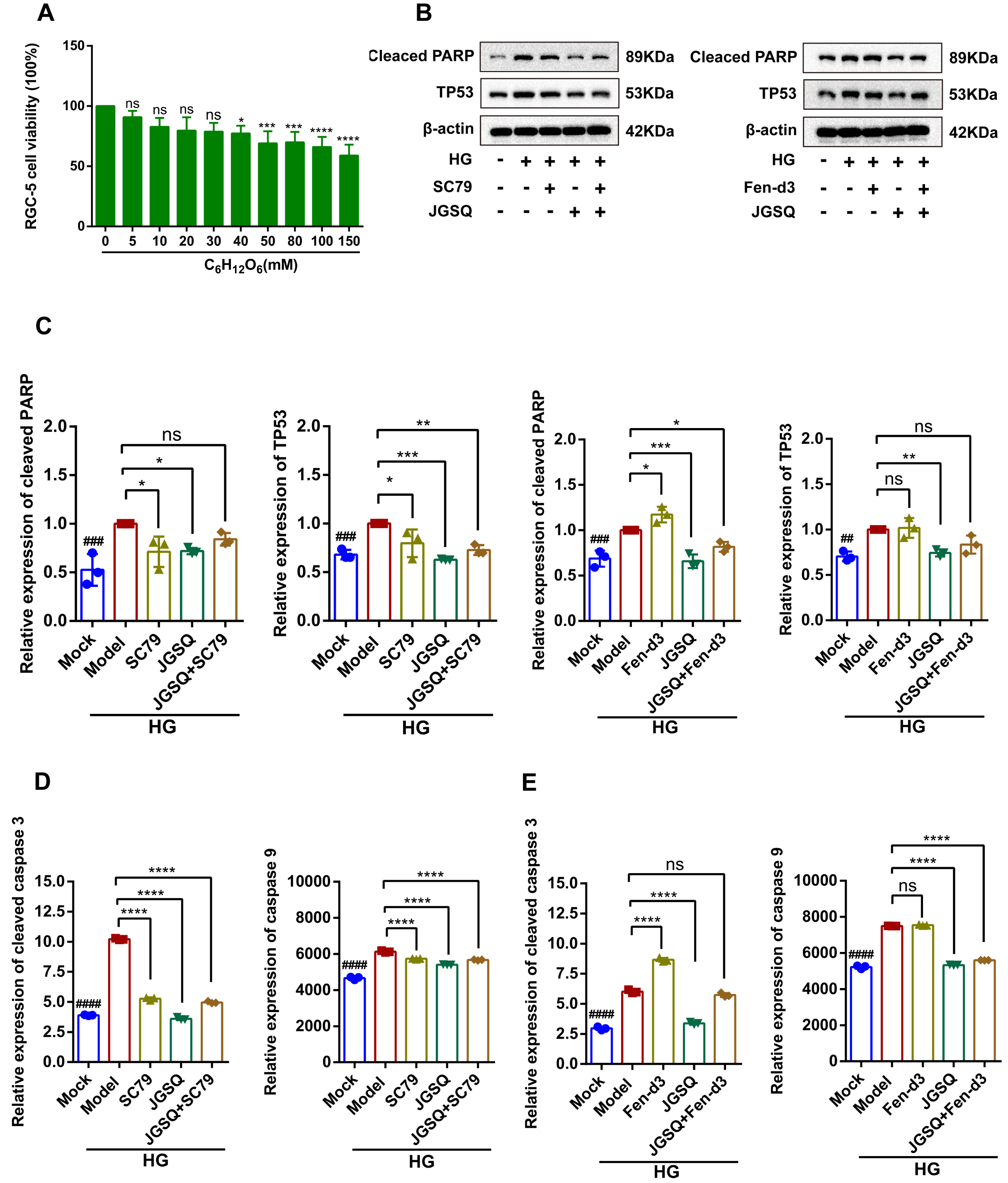

Supplement: Supplementary file 2 — Additional file 2: Figure S2. The target of JGSQ was validated in vitro. (A) Cell survival rate of RGC-5 cells under different concentrations of glucose. (B) The containing serum of JGSQ combined with the activators of AKT and HIF-1α respectively to alleviated the apoptosis of RGC-5 cells. (C) Statistical analyses of TP53 and cleaved PARP were performed using GraphPad Prism. (D-E) The levels of cleaved caspase 3 and caspase 9 in lysed RGC-5 cells were detected by ELISA kits. Groups VS Model.nsP >0.05, *P < 0.05, **P < 0.01, ***P < 0.001, ****P < 0.0001. AKT activator: SC79; HIF-1α activator: fen-d3 (fenbendazole-d3). [file 13020_2023_840_MOESM2_ESM.tif]
